# Supplementary figures and images for: Molecular basis for the role of disulfide-linked αCTs in the activation of insulin-like growth factor 1 receptor and insulin receptor
Source: eLife. 2022 Nov 22;11:e81286. doi: 10.7554/eLife.81286 (PMC9731570; doi:10.7554/eLife.81286)

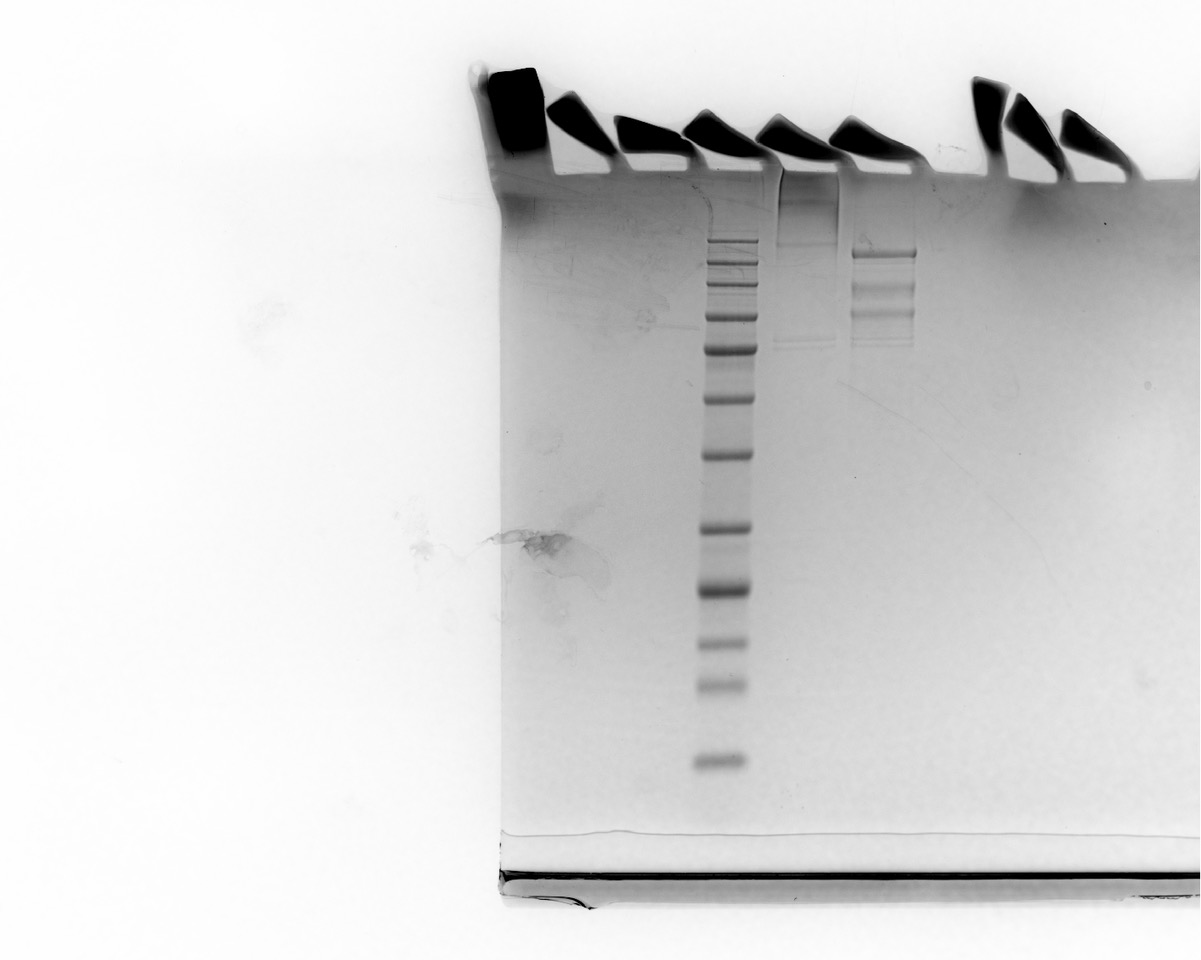

Supplement: Figure 1—figure supplement 2—source data 1. [file elife-81286-fig1-figsupp2-data1.zip › Figure 1-figure supplement 2-source data 1/IR.jpeg]

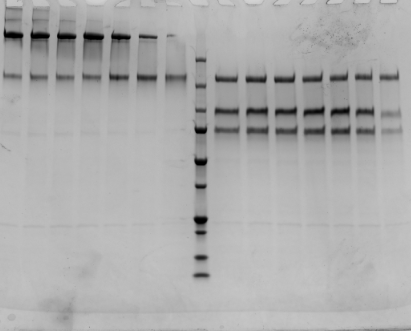

Supplement: Figure 1—figure supplement 2—source data 1. [file elife-81286-fig1-figsupp2-data1.zip › Figure 1-figure supplement 2-source data 1/IGF1R.png]

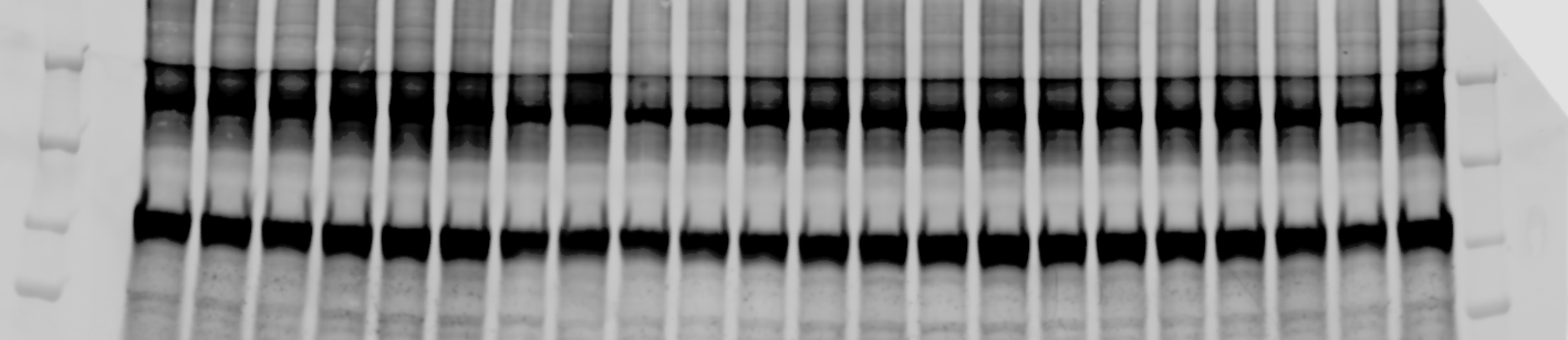

Supplement: Figure 3—source data 1. [file elife-81286-fig3-data1.zip › Figure 3-source data 1/Figure 3_IGF1R_total2.tif]

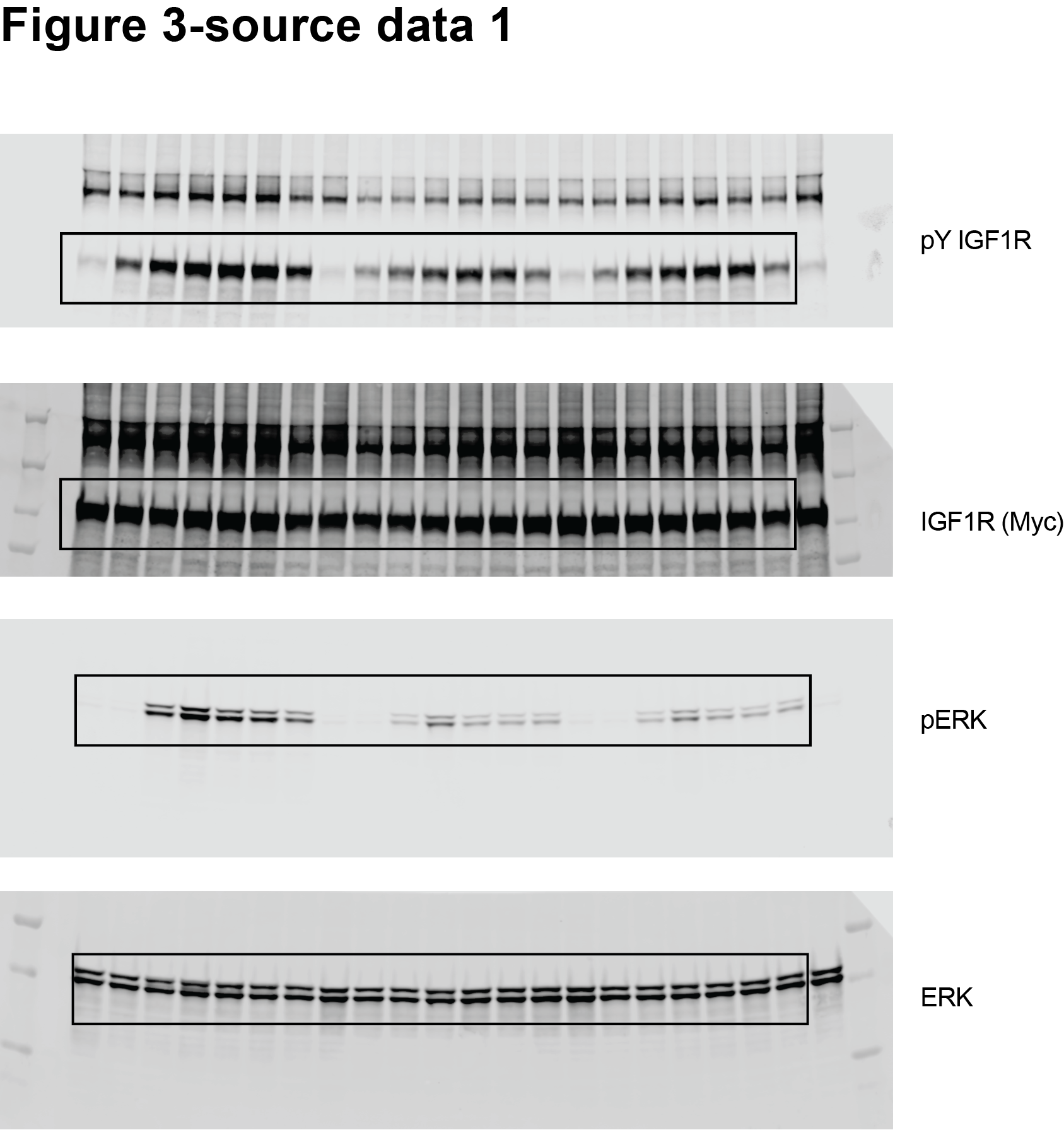

Supplement: Figure 3—source data 1. [file elife-81286-fig3-data1.zip › Figure 3-source data 1/Figure 3_source data 1_marker.png]

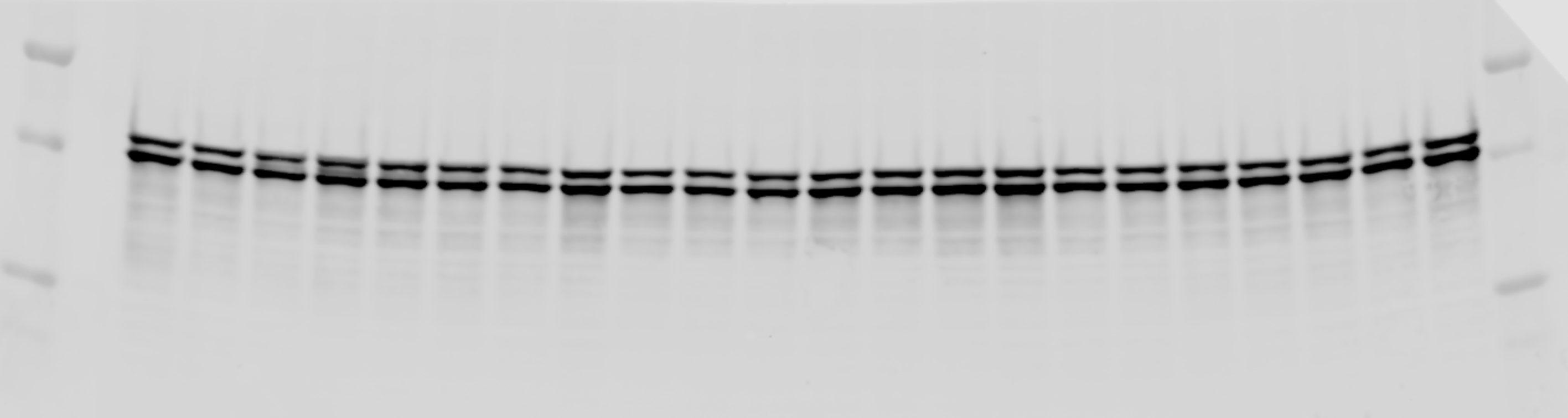

Supplement: Figure 3—source data 1. [file elife-81286-fig3-data1.zip › Figure 3-source data 1/Figure 3_ERK_total.tif]

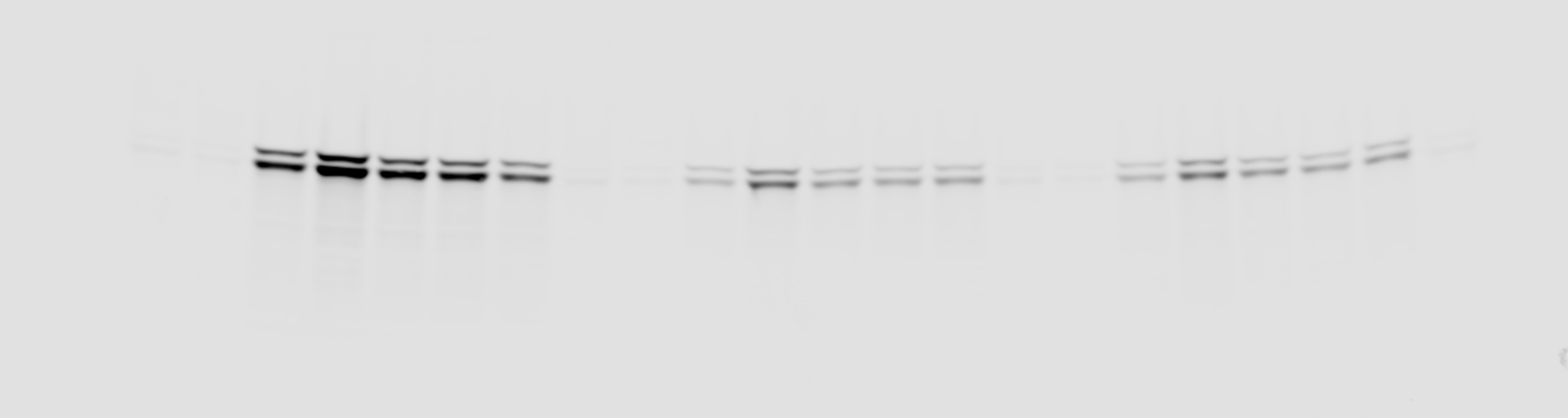

Supplement: Figure 3—source data 1. [file elife-81286-fig3-data1.zip › Figure 3-source data 1/Figure 3_pERK_p2.tif]

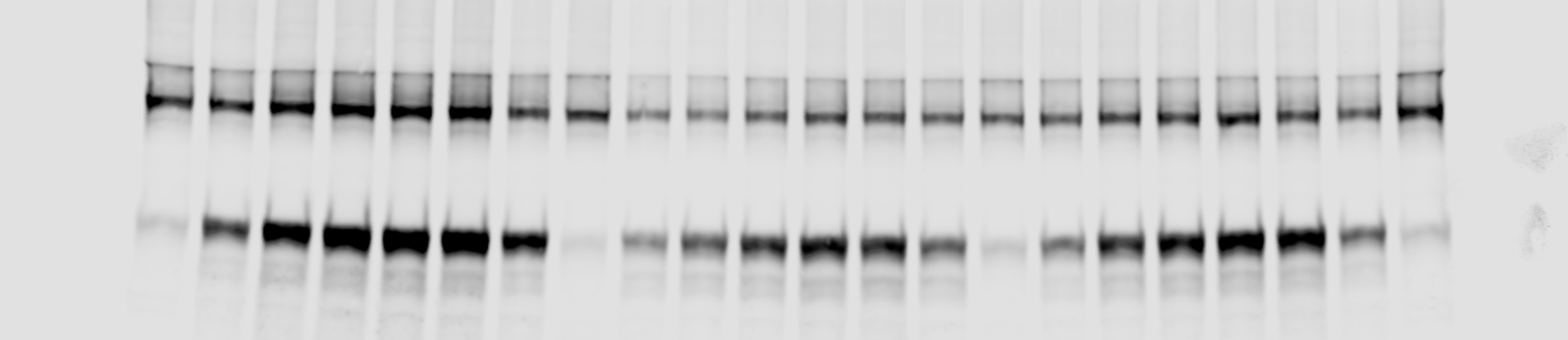

Supplement: Figure 3—source data 1. [file elife-81286-fig3-data1.zip › Figure 3-source data 1/Figure 3_pIGF1R_p2.tif]

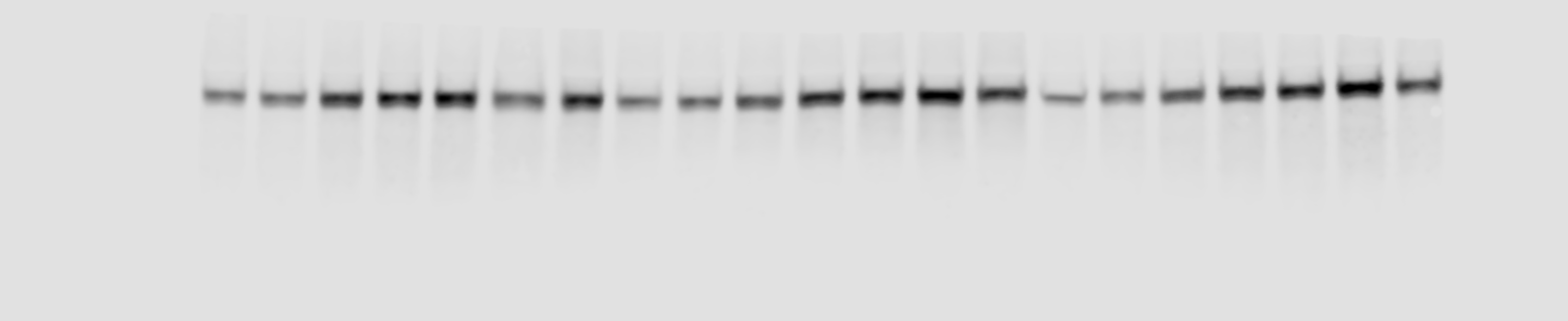

Supplement: Figure 6—source data 1. [file elife-81286-fig6-data1.zip › Figure 6-source data 1/Figure 6_pAKT.tif]

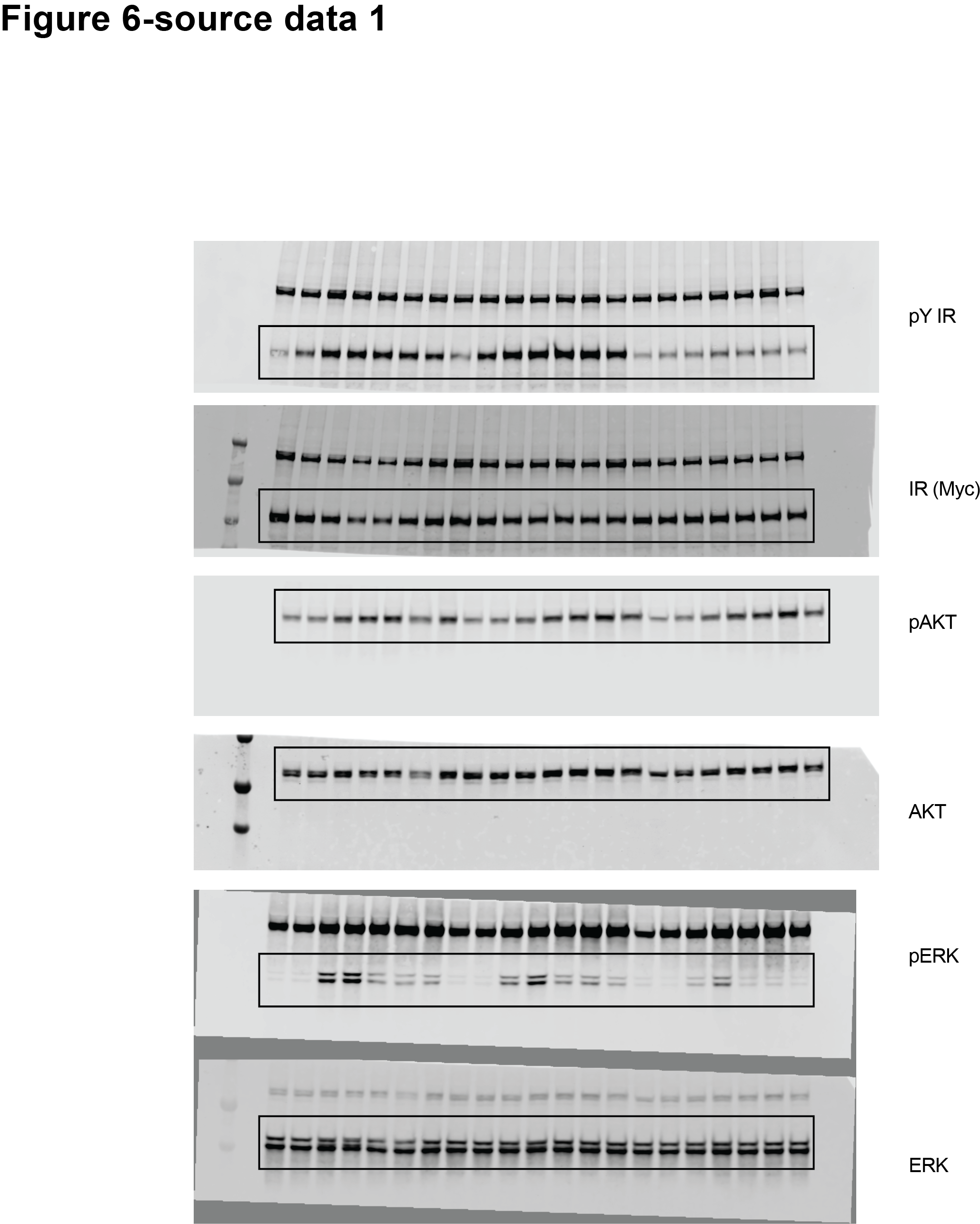

Supplement: Figure 6—source data 1. [file elife-81286-fig6-data1.zip › Figure 6-source data 1/Figure 6_source data 1_marker.png]

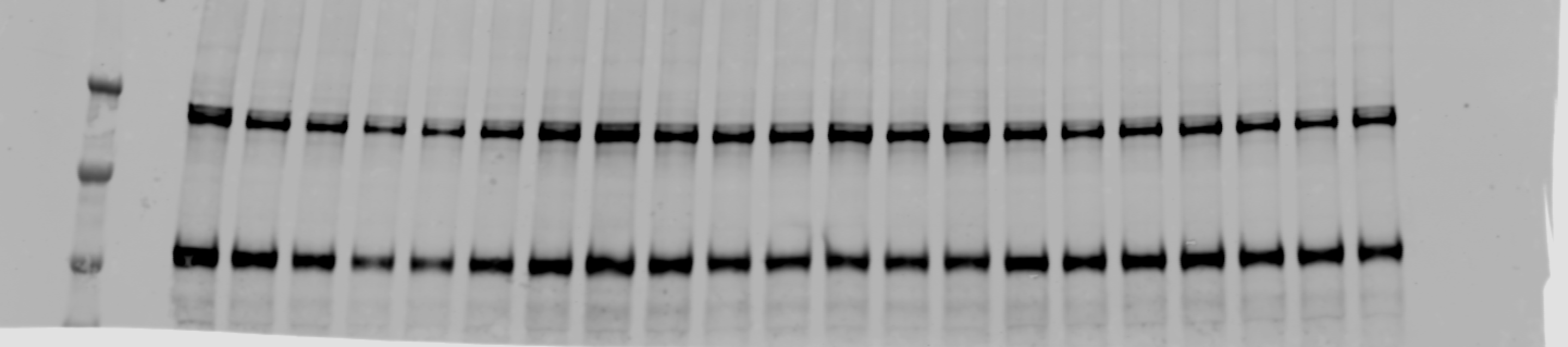

Supplement: Figure 6—source data 1. [file elife-81286-fig6-data1.zip › Figure 6-source data 1/Figure 6_IR_total.tif]

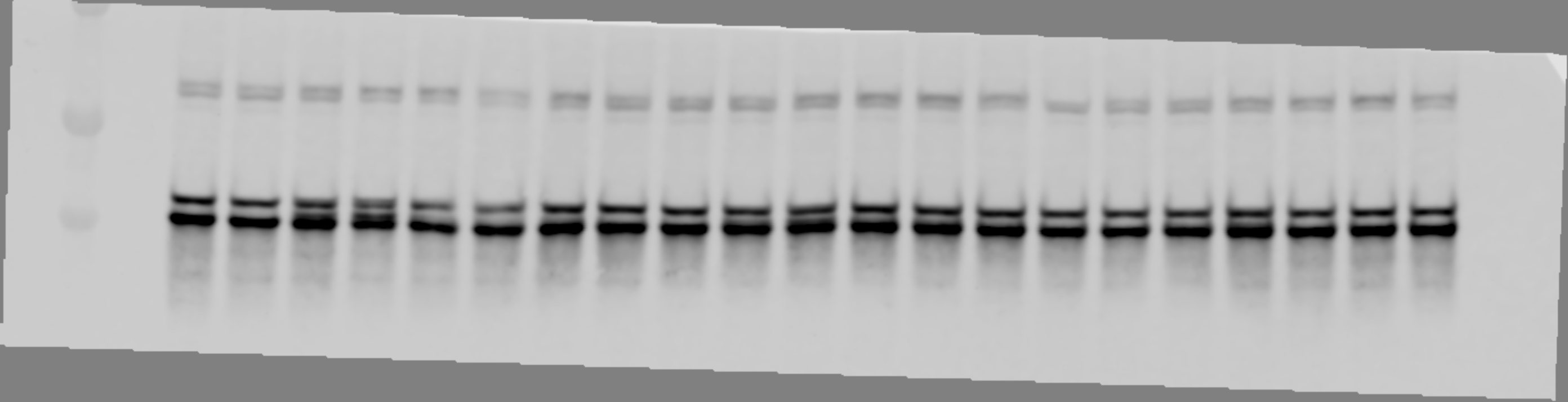

Supplement: Figure 6—source data 1. [file elife-81286-fig6-data1.zip › Figure 6-source data 1/Figure 6_ERK_total.tif]

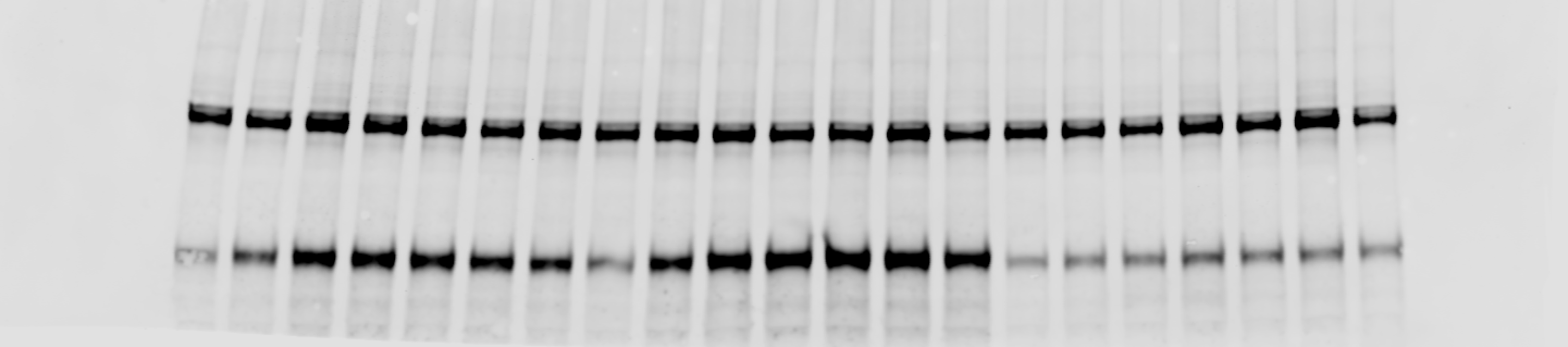

Supplement: Figure 6—source data 1. [file elife-81286-fig6-data1.zip › Figure 6-source data 1/Figure 6_pYIR.tif]

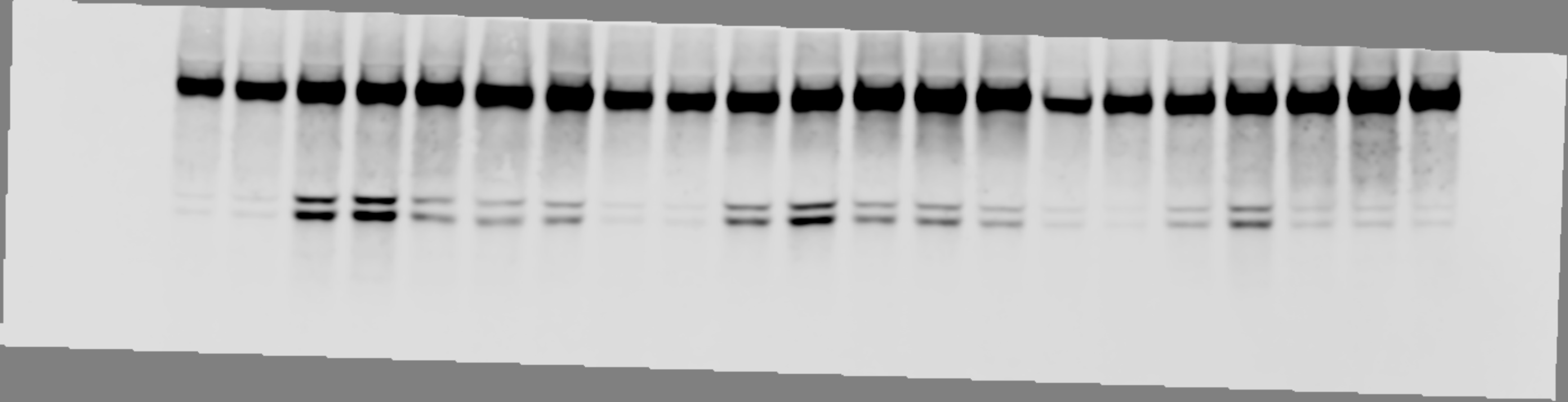

Supplement: Figure 6—source data 1. [file elife-81286-fig6-data1.zip › Figure 6-source data 1/Figure 6_pERK.tif]

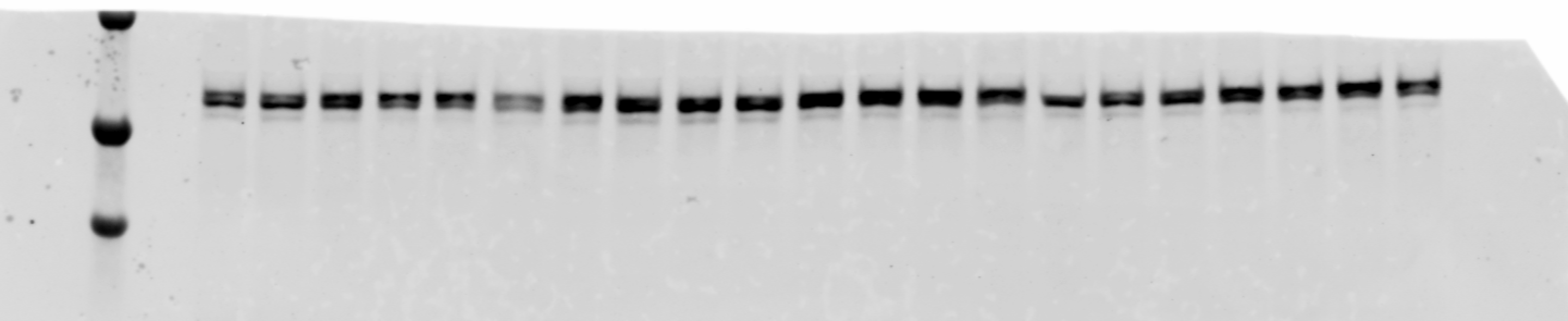

Supplement: Figure 6—source data 1. [file elife-81286-fig6-data1.zip › Figure 6-source data 1/Figure 6_AKT_total.tif]

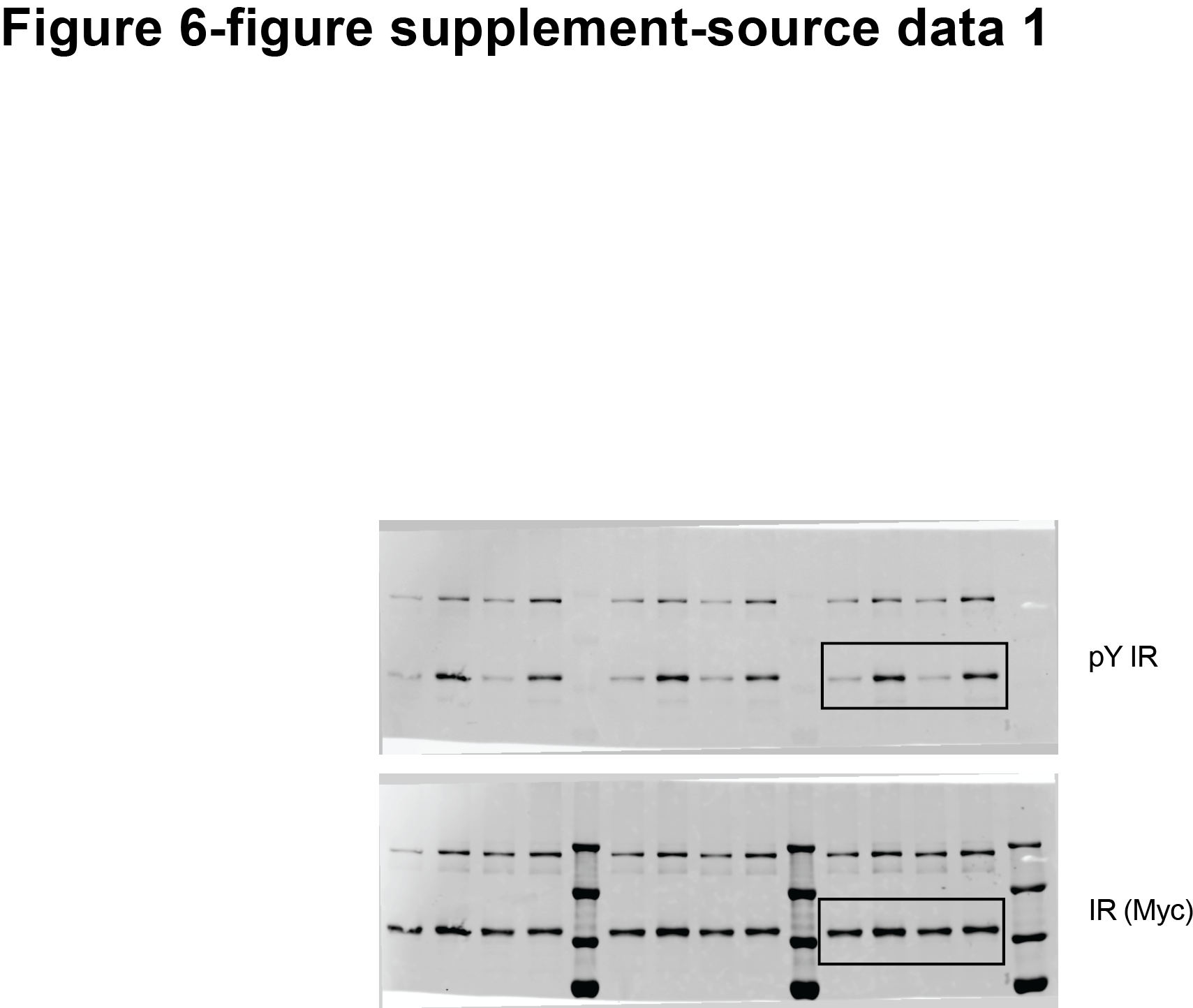

Supplement: Figure 6—figure supplement 1—source data 1. [file elife-81286-fig6-figsupp1-data1.zip › Figure 6-figure supplement 1-source data 1/Figure 6_figure supplement-source data 1.png]

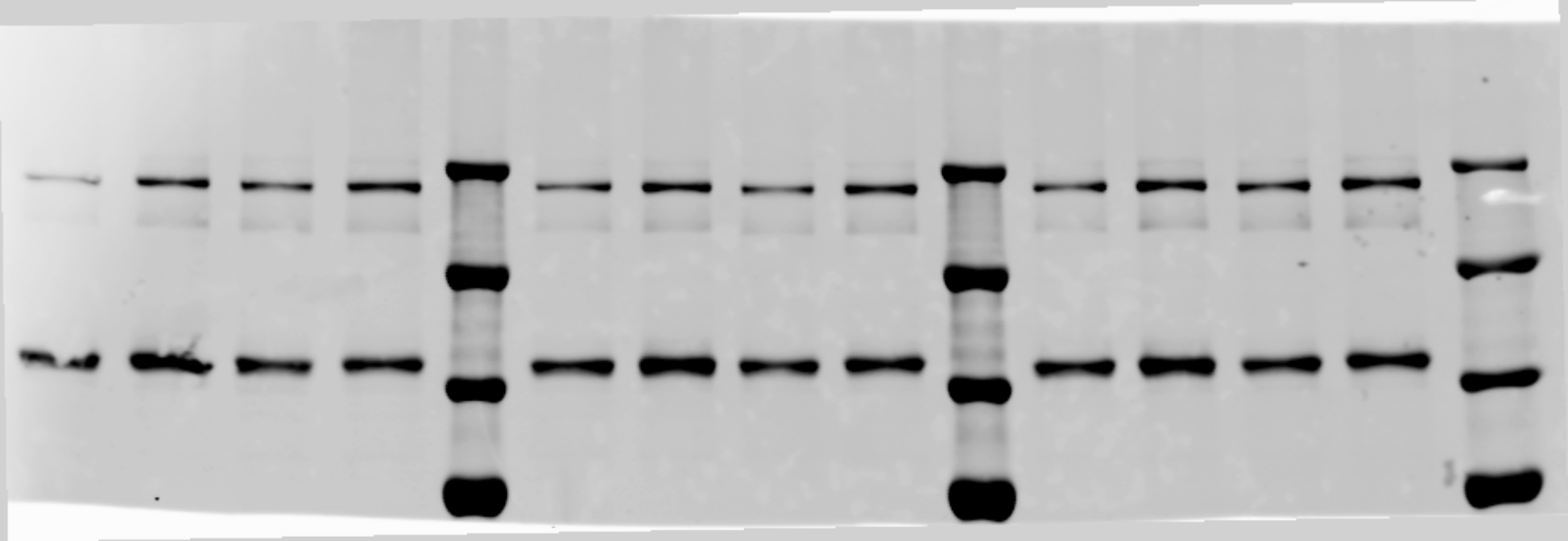

Supplement: Figure 6—figure supplement 1—source data 1. [file elife-81286-fig6-figsupp1-data1.zip › Figure 6-figure supplement 1-source data 1/IR_total.tif]

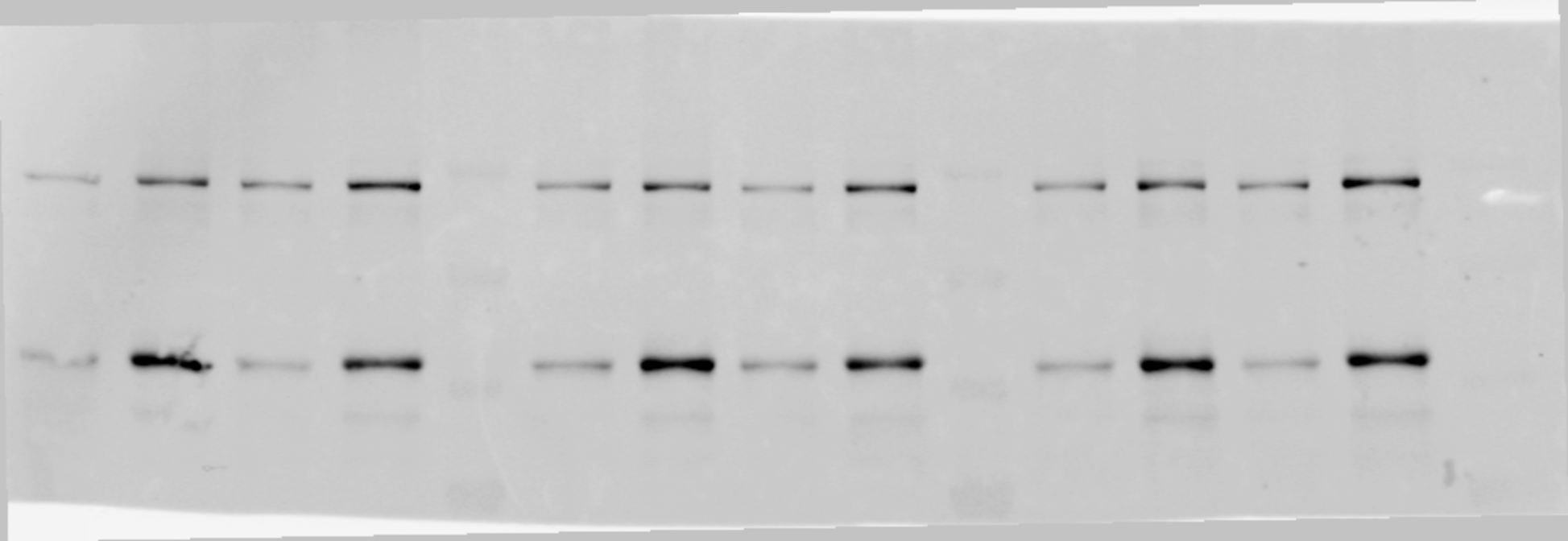

Supplement: Figure 6—figure supplement 1—source data 1. [file elife-81286-fig6-figsupp1-data1.zip › Figure 6-figure supplement 1-source data 1/pY IR.tif]
